# Kainate Receptor Antagonists: Recent Advances and Therapeutic Perspective

**DOI:** 10.3390/ijms24031908

**Published:** 2023-01-18

**Authors:** Paulina Chałupnik, Ewa Szymańska

**Affiliations:** Department of Technology and Biotechnology of Drugs, Jagiellonian University Medical College in Kraków, PL 30-688 Kraków, Poland

**Keywords:** glutamate ionotropic receptors, kainate receptors, antagonist, CNS diseases, neurological diseases, epilepsy, chronic pain

## Abstract

Since the 1990s, ionotropic glutamate receptors have served as an outstanding target for drug discovery research aimed at the discovery of new neurotherapeutic agents. With the recent approval of perampanel, the first marketed non-competitive antagonist of AMPA receptors, particular interest has been directed toward ‘non-NMDA’ (AMPA and kainate) receptor inhibitors. Although the role of AMPA receptors in the development of neurological or psychiatric disorders has been well recognized and characterized, progress in understanding the function of kainate receptors (KARs) has been hampered, mainly due to the lack of specific and selective pharmacological tools. The latest findings in the biology of KA receptors indicate that they are involved in neurophysiological activity and play an important role in both health and disease, including conditions such as anxiety, schizophrenia, epilepsy, neuropathic pain, and migraine. Therefore, we reviewed recent advances in the field of competitive and non-competitive kainate receptor antagonists and their potential therapeutic applications. Due to the high level of structural divergence among the compounds described here, we decided to divide them into seven groups according to their overall structure, presenting a total of 72 active compounds.

## 1. Introduction

Synaptic transmission is a fundamental form of transmission in the central nervous system (CNS), dependent on receptors that detect the released neurotransmitter and produce an appropriate and well-planned response. The general model of the organization and function of the CNS in the mammalian brain can be expressed as a specific coordinated balance between the excitatory and inhibitory systems, based on neurons that release glutamate (Glu) and γ-aminobutyric acid (GABA), respectively. Glu is an essential fast excitatory neurotransmitter involved in multiple neuronal functions such as learning, memory, and synaptic plasticity [1].

Until now, numerous glutamate receptors have been identified and initially divided into two main groups: metabotropic glutamate receptors (mGluRs) and ionotropic glutamate receptors (iGluRs), according to how the neurotransmitter modulates the receptor. The mGluR family belongs to class C of the G protein-coupled receptor (GPCR) superfamily. As such, they play a modulatory role in the regulation of neuronal and neurotransmitter excitability and are responsible for more prolonged stimulus effects by initiating signaling cascades and secondary messengers. The second group of glutamate receptors are ligand-gated cation channels, further divided into four functionally distinct subclasses named after their selective agonists: α-amino-3-hydroxy-5-methyl-4-isoxazolepropionic acid (AMPA) receptors, kainate (KA) receptors, *N*-methyl-d-aspartic acid (NMDA) receptors, as well as the little-known orphan δ (or GluD) receptors. The canonical mechanism of action in the case of iGluRs is related to the opening of the ion channel as a result of the binding of an agonist, allowing the flow of cations such as Na^+^, K^+^, and Ca^2+^, which ultimately leads to membrane depolarization and neuronal excitation [1,2,3].

The above delimitation between the mGluR and iGluR families is more complex from a functional point of view, considering that NMDA, AMPA, and kainate receptors, along with the classical mechanism of ligand-gated ion channels, also demonstrate metabotropic and non-canonical mechanisms of action [4,5].

## 2. Structure and Properties of Kainate Receptors

### 2.1. Structure of Ionotropic Kainate Receptors

The ionotropic glutamate receptor is a complex composed of four individual transmembrane subunits that form a central ion channel. Across the membrane, each subunit forms three transmembrane helixes and one reentrant loop, responsible for the channel architecture. The extracellular part of the subunit is arranged in two separate domains, an N-terminal domain (ATD) and a ligand binding domain (LBD), the latter responsible for binding glutamate and other agonists. The receptor is terminated by a C-terminal domain (CTD), which is most varied in the amino acid sequence. Functional iGluRs occur as heterotetramers composed of two pairs of subunits (dimers of dimers) or, more rarely, as homomers, and are formed exclusively by fusion with subunits within the same functional class of receptors. So-called “non-NMDA” receptors, namely AMPA receptors (AMPAR) and kainate receptors (KAR), can exist as homo- and heterotetramers within the same subfamily, while NMDA receptors (NMDAR) are obligate heterotetramers [2]. The known mammalian iGluR subunits are encoded by 18 genes and cluster into four subclasses: AMPA (GluA1-4, previously known as GluR1-4), KA (GluK1-5, previously GluR5-7, KA1, KA2), NMDA (GluN1, GluN2A-D, GluN3A, B), and δ (GluD1, GluD2) [2,6].

Kainate receptors (KARs), depending on their constituent subunits, were shown to vary in functional properties, as well as the affinity and potency of individual agonists for receptor activation and desensitization [7,8,9,10]. Subunits GluK1-3 (‘low-affinity’ subunits) can form either homomeric or heteromeric functional ion channels, and the corresponding homomeric GluK1-3 receptors show binding affinities for kainate in the range of 13–76 nM, while for (*S*)-glutamate in the range of 140–494 nM [9,11]. Interestingly, GluK3 receptors show significantly lower activation sensitivity for both agonists, compared to any other member of the non-NMDA receptor family [7]. The GluK4 and GluK5 subunits (‘high affinity’ subunits) form functional channels only in heteromeric assembly with one of the GluK1-3 subunits [12]; however, when expressed as non-functional homomeric receptors, they bind kainate and glutamate with a higher affinity than GluK1-3 [9,13,14].

Additional variability develops within the NTD and CTD domains of the KAR subunits, which undergo alternative RNA splicing. The extracellular N-terminal domain of GluK1 can exist as the GluK1_1_ or GluK1_2_ variant, while the C-terminal domain produces the variants GluK1a, GluK1b, GluK1c, and GluK1d. For the CTD domain of the GluK2 and GluK3 subunits, three (a, b, and c) and two variants (a and b), respectively, have been reported. Alternative splicing has been shown to affect cell surface receptor expression, interaction with proteins, and receptor localization in the CNS [14,15].

Structural variation of the kainate receptors could also be the result of post-transcriptional editing of mRNA in a specific region of the pore loop, known as the glutamine-arginine receptor (Q/R) site, with modification involving the conversion of a glutamine residue to arginine. This alteration affects the properties of KARs in terms of the reduction in permeability to Ca^2+^ ions and occurs in the case of the GluK1 and GluK2 subunits, but not in the GluK3 subunit. Therefore, GluK3-containing receptors exhibit atypical biophysical properties, lower sensitivity to glutamate, and due to their high permeability, a stronger intracellular polyamine blocking than any other AMPA/kainate receptor [16,17].

Currently available antisera against kainate receptor subunits allowed for the determination of KAR occurrence in multiple areas of the central nervous system: hippocampal interneurons, lateral amygdala, cerebral cortex, dorsal root ganglia, bipolar cells of the retina, and cerebellum [18,19]. GluK1 subunits are expressed mainly in the interneurons of the hippocampus and cortex, but also in the Purkinje cells and sensory neurons, and GluK2 subunits are present mainly in the principal cells of the hippocampus and cerebellum. GluK3 subunits occur primarily in the neocortex and dentate gyrus (DG) of the hippocampus, and GluK4 subunits in the CA3 pyramidal neurons, neocortex, dentate gyrus, and Purkinje cells. GluK5 is widely distributed throughout the CNS, and GluK2/5 heteromers, as the most abundant kainate receptors in the brain, can be found in most principal cells and various types of interneurons [14,20,21].

### 2.2. Activity of Kainate Receptors

In contrast to AMPA or NMDA receptors, which are located primarily in postsynaptic compartments, KARs are widespread in both post- and presynaptic regions. Presynaptic kainate receptors play an important role in the modulation of neurotransmitter release in various synapses and are involved in short- and long-term synaptic plasticity, including long-term potentiation (LTP) and long-term depression (LTD) [18,22,23,24].

In the ‘classical’ signaling mode of KARs, similar to other glutamatergic ionotropic receptors, the binding of an agonist leads to the opening of the ion channel, which contributes to membrane depolarization and synaptic signaling. Compared to AMPARs, KARs exhibit slower activation and deactivation kinetics depending on the co-expression of auxiliary proteins, which facilitate synaptic integration in interneurons and pyramidal cells [24,25,26,27]. This role is fulfilled by neuropilin and tolloid-like proteins (NETO) that form permanent complexes with the GluK1-3 subunits, regulating their functionality, expression in synapses, and kinetic properties. NETO1 occurs abundantly in the hippocampus and forms complexes with postsynaptic KARs in mossy fibers (MF)-CA3 synapses, while NETO2 is expressed primarily in the cerebellum. Furthermore, NETO2 interacts with the K-Cl cotransporter 2 (KCC2) and, therefore, affects its expression in the hippocampal neurons [15,27,28].

**Figure 1:**
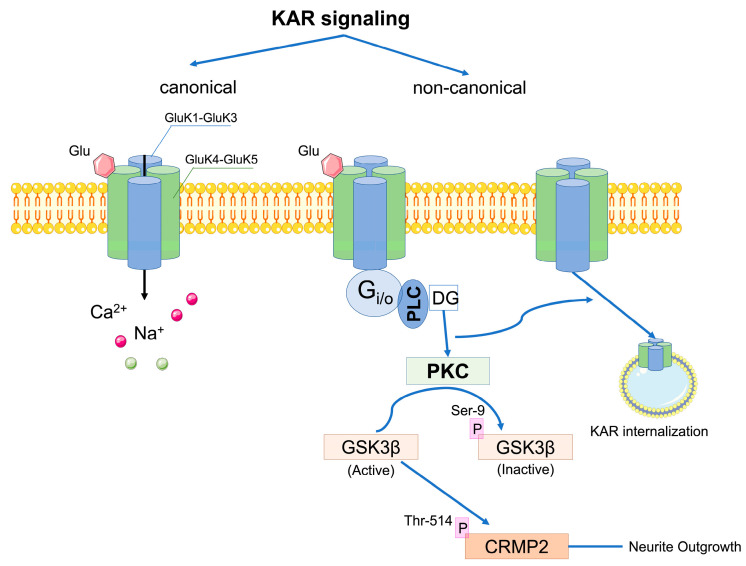
Canonical and non-canonical signaling of KARs (adapted from refs. [4,20]).

Presynaptically located kainate receptors have been implicated in the bidirectional modulation of both GABA and glutamate release and several mechanisms underlying these effects have been reported [4,5,29,30,31]. The observed inhibitory effect of KARs on GABA release in the axon terminals of the hippocampal interneurons seems to be independent of ion channel signaling and is related to the metabotropic mechanism involving G_i/o_ proteins, phospholipase C (PLC), and protein kinase C (PKC) (Figure 1). Since KARs do not interact directly with G proteins, it has been suggested that some kind of adapter protein may be involved in these interactions, although this has not been demonstrated so far [4,5,30]. Interestingly, a facilitation of GABA release mediated by kainate receptors activated by low concentrations of agonist has also been described [5,24]. Non-canonical signaling of presynaptic KARs similarly affects glutamate release, occurring in different synapses and being developmentally regulated. In the hippocampus, presynaptic KARs down-regulate glutamatergic transmission in Schaffer Collateral (SC)-CA1 synapses, while in MF-CA3 synapses, this regulation depends on KA concentration. Glutamate release is facilitated at low concentrations of the agonist, whereas it decreases at high nanomolar concentrations.

Postsynaptic kainate receptors are involved in synaptic transmission by mediating a small current with slow activation and deactivation kinetics. In the hippocampus, when activated by kainate or glutamate, KARs control neuronal excitability through the inhibition of hyperpolarization currents, resulting in a marked increase in the firing frequency of the CA1 pyramidal cell action potentials. This activity proceeds by coupling KARs to G_i/o_ and PKC proteins, indicating an additional metabotropic mechanism of action [5].

Kainate receptors also have the potential to affect the autoregulation of their membrane expression through activity-dependent internalization triggered by PKC phosphorylation. Furthermore, KARs are involved in the regulation of neurite growth in DG neurons, while the GluK2 subunit, through coexistence in a functional complex with the neuronal cotransporter KCC2, has an important role in the structural maturation of dendritic spines [4,32].

One of the suggested mechanisms involved in the metabotropic KAR pathway is related to the NMDAR-independent regulation of LTP in the CA1-CA3 synapses. Kainate-dependent LTP recruits endosomal vesicle recycling in spines, as well as triggering their increased outgrowth and maturation. Moreover, it affects the recycling of AMPA receptors and enhances their surface expression in CA1 hippocampal neurons [33]. Activation of kainate receptors may also affect the presynaptic form of LTP, e.g., at thalamocortical synapses and in the cortical input of the lateral amygdala [24].

**Figure 2:**
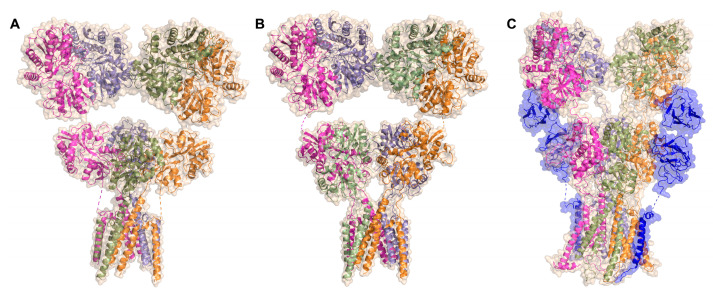
(**A**) Cryo-EM structure of the GluK2/5 heterotetramer in glutamate (l-Glu) bound state (PDB code: 7KS3); (**B**) cryo-EM structure of the GluK2/5 heterotetramer in the 6-cyano-7-nitroquinoxaline-2,3-dione (CNQX) bound state (PDB code: 7KS0); (**C**) cryo-EM structure of homotetrameric GluK2 in complex with NETO2 and DNQX, NETO2 shown in dark blue (PDB code: 7F5A).

Attempts to structurally resolve the architecture of tetrameric kainate receptors have led to low-resolution structures of the GluK3 receptor in complex with kainate or the antagonists UBP301 and UBP310 [34,35], along with cryo-EM structures of the heterotetramer associated with l-Glu or the antagonist CNQX (Figure 2) [36]. This allowed the characterization of changes occurring during the activation of the kainate receptor. It became clear that the heteromeric subunits of GluK2 and GluK5 participate quite differently in the channel activation and desensitization process, and the main structural rearrangements that involve peptide linkers connecting the pore helices occur in the GluK2 subunits [34,35,36,37]. Furthermore, recently reported cryo-EM structures of the homomeric GluK2 receptor in complex with one or two NETO2 subunits, in inhibited and desensitized states, revealed the molecular basis for the regulation of receptor gating and pore properties by the NETO2 protein [36].

### 2.3. Pathophysiological Role of Kainate Receptors

With the introduction of the first AMPAR/KAR antagonists into clinical trials in the 1990s, intense interest emerged in ionotropic glutamate receptors as therapeutic targets in numerous neurological disorders, including epilepsy. It is generally accepted that neuronal hyperexcitation and elevated glutamate concentrations in extracellular fluid play an important role in the induction of epileptic seizures, and it has been observed in rodent animal models that NMDAR, AMPAR, and KAR agonists can induce seizures, while antagonists suppress them.

The conviction that NMDA receptors play a key role in seizure generation has led to the development of NMDAR blocking agents that entered clinical studies for epilepsy [38]. However, the majority of NMDA receptor antagonists have been shown to be ineffective against fully kindled seizures and cause side effects such as hyperlocomotion and motor stereotypies. The exception with anticonvulsant activity includes ketamine, a non-competitive NMDAR antagonist applied in analgesia, whose anticonvulsant properties have been well proven [39,40]. Due to its adverse side effects, ketamine is currently administered to children with severe refractory epilepsy (Lennox–Gastaut syndrome and pseudo-Lennox syndrome) [39]. On the other hand, clinical trials of AMPAR antagonists have shown significant anticonvulsant efficacy among patients with partial-onset seizures and in the treatment of refractory status epilepticus, although they can also induce transient sedation.

These results led to a major breakthrough and the discovery of perampanel, the first marketed non-competitive AMPA receptor antagonist, for the treatment of focal seizures with/without secondary generalization or primary generalized tonic–clonic seizures [41,42].

Many studies demonstrate a clear connection between kainate receptors and epilepsy [43,44,45,46,47]. In the hippocampus of patients with this condition, enhanced expression of KARs has been found [46,48]. It should be noted that kainate itself is a highly potent neurotoxin that, after systemic and intracerebral administration to animals, triggers persistent seizures and paroxysmal brain injury syndrome, as well as neurodegeneration in the striatum and hippocampal region. Most research on epilepsy related to kainate receptor transmission uses kainate to induce behavioral and electrophysiological seizures, establishing an acute temporal lobe epilepsy model (TLE) [46,49]. Due to reduced GABA release in the presence of kainate, hippocampal pyramidal cells are suppressed, causing acute epileptic seizures. The application of selective GluK1-antagonists prevented epileptic activity, clearly demonstrating the key role of synaptic inhibition in KA-induced seizures. On the other hand, studies on a pilocarpine model of chronic TLE showed that interictal events and seizures were significantly reduced in GluK2 knock-out mice or in wild-type mice after the administration of the GluK2/5 antagonist [18,44,46]. All these observations clearly indicate that KARs play a significant role in ictogenesis and may constitute a biological target for epilepsy therapy [18,39,46].

On the other hand, low doses of kainate can affect the survival and growth of the neurites in the cerebellum, hippocampus, and spinal cord [4]. Studies on domoic acid neurotoxicity suggest that kainate receptor antagonists may also be effective in the neuroprotection of neurons and glial cells in neurodegenerative diseases such as Alzheimer’s disease, Huntington’s disease, and multiple sclerosis, or may also provide protection in models of global and focal ischemia [50,51].

The expression of kainate receptors has also been detected along pain neurons, including the dorsal root ganglion, the dorsal horn of the spinal cord, the thalamus, and the cortex, affecting nociceptive transmission and pain modulation [52,53,54]. Pharmacological studies support the efficacy of GluK1 receptor antagonists in the antinociceptive response in acute pain and chronic pain models, especially compared to selective antagonists of the AMPA receptor [52,54,55,56]. Subsequently, neurogenic dural vasodilation as an established model of trigeminal activation using the selective GluK1 antagonist LY466195 demonstrated a direct effect of these subunits on migraine [55,57,58]. The association of presynaptic GluK2/3 receptors that facilitate the release of GABA from some nerve terminals in the brain stem, with pain transmission, was also reported [59].

Genetic and postmortem studies of human brains have demonstrated the contribution of kainate receptors in the pathogenesis of several mental illnesses, including schizophrenia, bipolar disorder, depression, autism, and obsessive–compulsive disorder. Among others, reduced expression in the GluK1-3 subunits has been found in the orbitofrontal cortex of patients with schizophrenia. Several genetic studies have linked polymorphic variants of the GluK2 subunit to schizophrenia, autism, and obsessive–compulsive disorder, while variants of the GluK3 subunit have been linked to schizophrenia, bipolar disorder, and depression [60,61,62,63,64]. Recent results demonstrate the involvement of a heteromeric combination GluK2/5 in the expression of depressive behavior and the development of contextual conditional memory [36,65].

Understanding the exact function and therapeutic potential of kainate receptors has not reached a stage as advanced as that of AMPA or NMDA receptors, mainly due to the relative lack of specific pharmacological tools. Due to the high homology of orthosteric binding site regions observed between the AMPAR and KAR subunits, only a few pharmacological tools are known to distinguish between these two subfamilies of iGluR. Kainate itself, along with kainate receptors, also activates AMPA receptors as a weak partial agonist with a micromolar affinity [66,67].

In recent decades, intensive efforts have been made to develop non-NMDAR antagonists; however, only a limited number of them showed a high degree of selectivity in KA receptors over AMPA receptors. The development of highly selective antagonists of individual KAR subunits is even more problematic, and until now mainly GluK1-selective compounds have been reported.

## 3. Competitive Antagonists of the Kainate Receptors

### 3.1. Quinoxaline-2,3-diones

**Figure 3:**
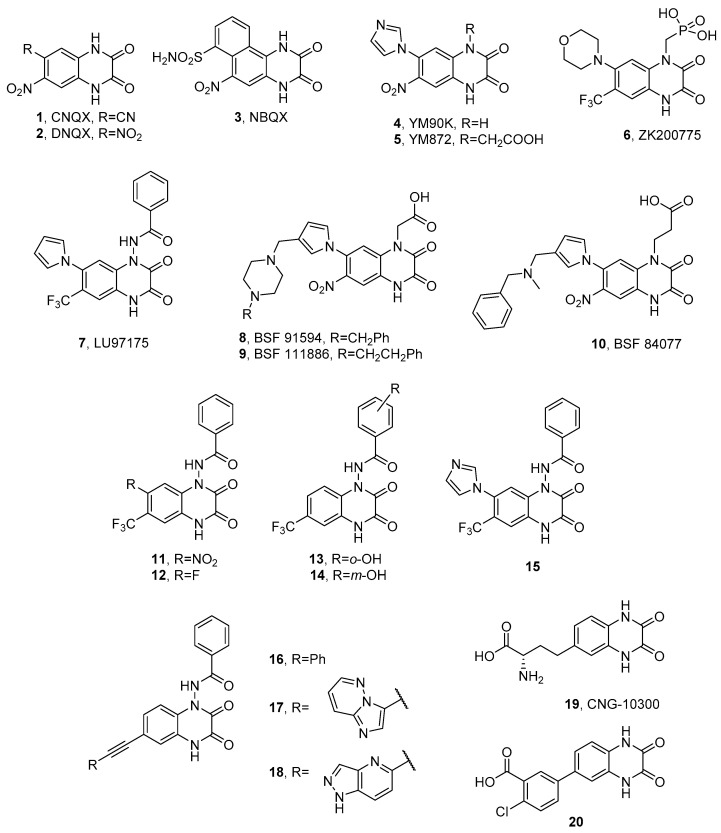
Structures of selected KAR antagonists based on the quinoxaline-2,3-dione structure.

Quinoxaline-2,3-dione analogues are one of the most intensively studied classes of competitive AMPAR/KAR antagonists. The early generation of quinoxalinediones, such as CNQX (**1**, Figure 3), DNQX (**2**), or NBQX (**3**), was reported in the 1980s and 1990s as potent AMPAR/KAR antagonists and neuroprotective agents [68,69,70,71,72]. Many members of this class also showed effects at the glutamate and, particularly, the glycine (GlyN) binding sites of the NMDA receptor. Among them, NBQX was the first compound to exhibit a high selectivity for AMPAR versus NMDAR (Table 1) and has been used as the referential antagonist of choice in numerous in vitro and in vivo models. The neuroprotective, anticonvulsant, anxiolytic, and antinociceptive effects of NBQX have been widely investigated and reported [69,73,74,75,76,77,78,79,80,81]. Neuroprotective properties of NBQX in ischemic stroke were confirmed in clinical studies; however, due to the low aqueous solubility and associated nephrotoxicity at therapeutic doses, it was rejected in the second phase of clinical studies [82].

The subsequent years progressed with numerous studies aimed at improving the affinity and selectivity toward AMPA receptors, the results of which have been reviewed in detail by Nikam [83], Catarzi [82], and Larsen [84]. Among them, particularly notable are YM90K (**4**), YM872 (**5**), and ZK200775 (**6**), active and selective AMPA receptor antagonists, also showing a micromolar binding affinity at the native kainate receptors (Table 1) [85,86,87,88]. Relevant structural modifications improved the affinity of the compounds of the AMPAR and solved the problem of low water solubility. Both YM872 and ZK200775 have demonstrated neuroprotective activity in animal models of acute and ischemic stroke and have become the subject of clinical trials [85,86,87,88]. Furthermore, YM872 showed neuroprotective properties in brain hemorrhage, as well as therapeutic potential in malignant primary glioma [89,90].

**Table 1:** Receptor binding affinity of early quinoxaline-2,3-diones at native iGluRs, determined in radioligand binding assays (all values in μM).

cmpd	Native iGluRs		
	AMPA [^3^H]AMPA	KA [^3^H]KA	NMDA [^3^H]CPP or [^3^H]Glu
CNQX (**1**)	0.30 ± 0.15 (IC_50_) *^a^* 0.25 ± 0.01 (*K*_i_) *^b^*	1.5 ± 0.3 (IC_50_) *^a^* 2.7 ± 0.1 (IC_50_) *^b^*	25 (IC_50_) *^a^* 45 ± 4 (IC_50_) *^b^*
DNQX (**2**)	0.50 ± 0.10 (IC_50_) *^a^*	2.0 ± 0.1 (IC_50_) *^a^*	40 (IC_50_) *^a^*
NBQX (**3**)	0.046 ± 0.001 (*K*_i_) *^b^* 0.15 ± 0.01 (IC_50_) *^c^* 0.12 ± 0.04 (*K*_i_) *^d^*	6.8 ± 0.2 (IC_50_) *^b^* 4.8 ± 0.5 (IC_50_) *^c^* 3.7 ± 1.0 (*K*_i_) *^d^*	>100 (IC_50_) *^b^* >90 (IC_50_) *^c^*
YM90K (**4**)	0.071 ± 0.002 (*K*_i_) *^b^*	4.4 ± 0.1 (IC_50_) *^b^*	>100 (IC_50_) *^b^*
YM872 (**5**)	0.096 ± 0.002 (*K*_i_) *^b^*	4.6 ± 0.1 (IC_50_) *^b^*	>100 (IC_50_) *^b^*
ZK200775 (**6**)	0.12 ± 0.09 (IC_50_) *^e^*	2.5 ± 0.2 (IC_50_) *^e^*	2.8 ± 0.35 (IC_50_) *^e^*

*^a^* ref. [68], NMDA radioligand (the orthosteric NMDAR binding site): [^3^H]3-(2-carboxypiperazin-4-yl)propyl-1-phosphonic acid ([^3^H]CPP); *^b^* ref. [91], NMDA radioligand [^3^H]Glu; *^c^* ref. [69], NMDA radioligand: [^3^H]CPP; *^d^* ref. [72]; *^e^* ref. [88], NMDA radioligand: [^3^H]CPP.

Selectivity toward KA receptors over AMPA receptors has been shown for pyrrolylquinoxaline-2,3-dione analogues, including the highly potent LU97175 (**7**, Figure 3), the compound substituted with a benzhydrazide group at the *N*1 position of the quinoxalinedione core [72]. Interestingly, LU97175 was reported to preferentially bind to the GluK3 homomeric receptors compared not only to the AMPA subtype GluA2, but also to the other individual kainate subtypes GluK1, GluK2, and GluK5 (Table 2) [72,92]. In an in vivo kindling model of temporal lobe epilepsy, LU97175 displayed anticonvulsant activity without inducing motor damage [71,72,93].

Among the examples of highly selective antagonists for homomeric GluK1 receptors are pyrrolyl quinoxalinediones BSF 91594 (**8**), BSF 111886 (**9**), and BSF 84077 (**10**), possessing an aliphatic amine (e.g., piperazine) linker attached to the pyrrole ring (Figure 3). Compounds **8** and **9** were found to be potent GluK1 ligands with more than a 100-fold selectivity over other kainate subtypes (Table 2). All three compounds showed a moderate to weak affinity at the native AMPA receptors and weak or no binding to the NMDA glycine binding site. When tested in vivo, **8**, **9**, and **10** confirmed their anticonvulsant potency in NMDA-induced seizures with effective median doses of 14, 21, and 3.3 mg/kg, respectively [94].

The LU97175 structure became the starting point for further modifications of the quinoxalinedione scaffold at positions *N*1, 6, and 7. SAR studies for the recently reported series of *N*1-substituted analogues confirmed that, also for compounds without a pyrrole ring in position 7 (and possessing a fluorine or nitro group instead), the *N*1-benzamide moiety was optimal for binding to the GluK1-3 recombinant receptors (**11**–**14**) and sufficient to achieve an affinity profile similar to LU97175 (**12**), while compounds with smaller or non-aromatic substituents in position *N*1 showed a significantly lower KAR affinity (Table 2) [92]. Attempts to bioisosterically replace the pyrrolyl moiety in the structure of LU97175 resulted in another series of derivatives, among which a 7-imidazole analogue (**15**) exhibited the highest binding affinity across the recombinant receptors GluK1-3 and the 73-fold binding selectivity for GluK3 over the AMPA subtype GluA2 (Table 2) [95]. In a mouse tail flick test for acute pain, **15** was shown to have an analgesic effect and was more effective than NBQX, suggesting that, contrary to an earlier hypothesis [75], the analgesic effects observed for early generation quinoxalinediones could also be mediated by kainate receptors. Furthermore, **15** did not show an adverse effect on motor coordination in the rotarod test as previously observed for NBQX, and it was found to cause an increase in locomotor activity [95].

The binding mode of **15** in the GluK1 binding pocket was determined by X-ray crystallography, showing a strong analogy to other complexes of quinoxaline-2,3-diones cocrystallized with the LBDs of non-NMDA receptors. Among others, **15** forms hydrogen bonds between the carbonyl groups of the quinoxalinedione core and Arg523 of domain 1, the residue considered crucial for the binding of l-glutamate, agonists, and competitive antagonists, and conserved within all AMPA and KA subunits [11,96]. The quinoxaline-2,3-dione core is also involved in H-bond contacts with other residues important for ligand binding: Thr518 and Pro516, while the position of the molecule is stabilized by π−π stacking between the quinoxaline-2,3-dione system and Tyr489 [95]. Despite the fact that **15** does not form any direct hydrogen bond with the residues of domain 2, the ligand, similarly to other quinoxalinediones, induces the opening of the LBD consistent with an antagonist binding mode. Analysis of the **15**-GluK1-LBD X-ray structure also revealed the presence of a sulfate ion in the ligand-binding site. Considering that the unsubstituted imidazole nitrogen atom of **15** is probably protonated (the complex was crystallized at pH 4.5), it likely forms a salt bridge with a sulfate ion, which could explain the higher activity of this compound compared to LU97175 [95].

The *N*1-benzamide analogue **16** with an unsubstituted 7-position of the quinoxalinedione scaffold and possessing a phenylethynyl moiety at position 6 (Figure 3), demonstrated an interesting selectivity profile showing approximately a 30-fold preference for the GluK3 homomeric receptors over the subtypes GluK1 and GluK2 [92]. Following **16** as the lead structure, a series of quinoxaline-2,3-dione analogues with an ethynyl substituent at the 6-position has recently been presented [97]. The introduction to this position of a large bicyclic substituent linked by a triple bond to the quinoxalinedione core (**17**, **18**) appeared to be an important structural modification that clearly affected the binding to the GluK3 subunit, resulting in the compound **17** with a pronounced preference for GluK3 and submicromolar GluK3 affinity (*K*_i_ = 0.253 μM, Table 2). In fact, a 400-fold selectivity for GluK3 over the subtypes GluK1, GluK2, GluK5, and GluA2, reported for this analogue, has been a unique KA receptor affinity profile among all structures described to date [97]. On the other hand, the most active compound in this series, **18**, showed a weak selectivity profile but a high affinity at the homomeric receptors GluK1, GluK3, and also GluK2, thus presenting one of the highest GluK2-affinity values among the quinoxalinedione-based ligands reported so far (Table 2).

**Table 2:** Receptor binding affinity of selected quinoxaline-2,3-diones at recombinant homomeric iGluRs, determined in radioligand binding assays (all values in μM).

cmpd	*K*_i_ [μM]				
	Recombinant Homomeric iGluRs				
	GluK1	GluK2	GluK3	GluK5	GluA2
CNQX (**1**)	1.3 ± 0.3 *^a^*	1.1 ± 0.1 *^b^* 1.5 ± 0.01 *^a^*	0.64 ± 0.05 *^a^*	8.4 ± 0.9 *^c,d^*	0.33 ± 0.03 *^a^*
DNQX (**2**)	0.65 ± 0.03 *^a^*	2.1 ± 0.3 *^a^*	0.36 ± 0.03 *^a^*	7.1 ± 0.9 *^a^*	0.25 ± 0.01 *^a^*
NBQX (**3**)	1.4 ± 0.4 *^e^* 12 ± 4 *^f^* 2.6 ± 0.1 *^d^*	2.3 *^e^* 13 ± 2 *^f^* 5.4 ± 1.2 *^d^*	3.2 *^e^* 24 ± 7 *^f^* 18 ± 8 *^b^* 3.4 ± 0.6 *^d^*	19 ± 8 *^e^* >100 *^f^* >100 *^c^* 152 ± 23 *^d^*	0.26 ± 0.04 *^f^* 0.077 ± 0.010 *^d^*
LU97175 (**7**)	0.088 ± 0.033 *^e^* 0.70 ± 0.12 *^d^*	0.31 ± 0.19 *^e^* 0.49 ± 0.06 *^d^*	0.022 ± 0.003 *^e^* 0.19 ± 0.029 *^d^*	6.9 ± 0.03 *^e^* 24 ± 4 *^d^*	1.52 ± 0.25 *^d^*
**8** *^g^*	0.0038	4.1	0.68	20	nd
**9** *^g^*	0.012	1.4	1.4	25	nd
**10** *^g^*	0.43	23	nd	>30	nd
**11** *^d^*	1.1 ± 0.1	2.0 ± 0.1	0.53 ± 0.15	43 ± 11	4.9 ± 0.6
**12** *^d^*	0.80 ± 0.11	0.81 ± 0.08	0.28 ± 0.03	31 ± 4	6.2 ± 1.4
**13** *^d^*	1.1 ± 0.1	0.91 ± 0.17	0.14 ± 0.02	26 ± 2	4.1 ± 0.6
**14** *^d^*	0.80 ± 0.18	0.84 ± 0.19	0.33 ± 0.01	45 ± 4	6.0 ± 0.1
**15** *^h^*	0.17 ± 0.01	0.52 ± 0.14	0.08 ± 0.01	5.2 ± 0.30	5.7 ± 0.3
**16** *^d^*	>100	≈100	2.9 ± 0.3	>100	24 ± 6
**17** *^i^*	≈100	>100	0.25 ± 0.01	>100	>100
**18** *^i^*	0.15 ± 0.05	0.091 ± 0.01	0.13 ± 0.03	3.8 ± 0.1	0.23 ± 0.02
**19** *^a^*	16 ± 1	9.5 ± 1.2	59 ± 3	nd	21 ± 2
**20** *^j^*	1.2 ± 0.7	33 ± 3	37 ± 4	nd	>100

*^a^* ref. [98], radioligands: GluA2, [^3^H]AMPA; GluK1-3, [^3^H](2*S*,4*R*)-4-methyl-Glu ([^3^H]SYM2081) or [^3^H]KA; *^b^* ref. [99], radioligand: [^3^H]KA; *^c^* ref. [13], radioligand: [^3^H]KA; *^d^* ref. [92], radioligands: GluA2, [^3^H]AMPA; GluK1, [^3^H]KA or (*S*)-2-amino-3-(6-[^3^H]-2,4-dioxo-3,4-dihydrothieno [3,2-d]pyrimidin-1(2*H*)-yl)propanoic acid ([^3^H]-(*S*)-NF608); GluK2,3,5, [^3^H]KA; *^e^* ref. [72], radioligand: [^3^H]KA; *^f^* ref. [100], radioligands: GluA2, [^3^H]AMPA; GluK1-3,5 and GluK5, [^3^H]KA; *^g^* ref. [94], radioligand: [^3^H]KA; *^h^* ref. [95], radioligands: GluA2, [^3^H]AMPA; GluK1, [^3^H]-(*S*)-NF608; GluK2,3,5, [isopropenyl-^3^H]-kainic acid; *^i^* ref. [97], radioligands: GluA2, [^3^H]AMPA; GluK1, [^3^H]-(*S*)-NF608; GluK2,3,5, [isopropenyl-^3^H]-kainic acid; *^j^* ref. [101], radioligands: GluA2, [^3^H]AMPA; GluK1, [^3^H]-(*S*)-NF608; GluK2,3, [^3^H]KA; nd—not determined.

Demmer et al. presented a different approach to the design of quinoxalinedione structures [98,101]. The proposed modification focused on the combination of an unsubstituted quinoxaline-2,3-dione core with an amino acid chain (**19**) [98] or an acid moiety, varied in chemical functionalities, carbon chain length, and flexibility (**20**) [101]. The amino acid derivative CNG-10300 (**19**) exhibited a weak micromolar affinity for homomeric GluK1-3 receptors with *K*_i_ values of 16, 9.5, and 59 μM, respectively, and was successfully cocrystallized with the GluK1-LBD. Analysis of this X-ray complex indicated that the amino acid moiety of **19** did not interact with the protein in the way observed for typical α-amino acid agonists but was involved in interactions that stabilized an open antagonist state of the binding pocket [98]. Micromolar binding to GluK1-3 was also observed among the series of compounds containing an acidic group. The most active kainate ligand, **20**, with an affinity of 1.2 µM for the GluK1 homomeric receptors showed more than a 27-fold selectivity over the kainate subtypes GluK2 and GluK3, and a 20-fold selectivity over the native AMPA receptors [101]. In a broader sense, the weak KAR affinity of the described monosubstituted quinoxalinediones confirmed the need for an additional core substitution in *N*1 to achieve a high affinity for the kainate receptors.

### 3.2. α-Amino Acid Antagonists

#### 3.2.1. Willardiines

**Figure 4:**
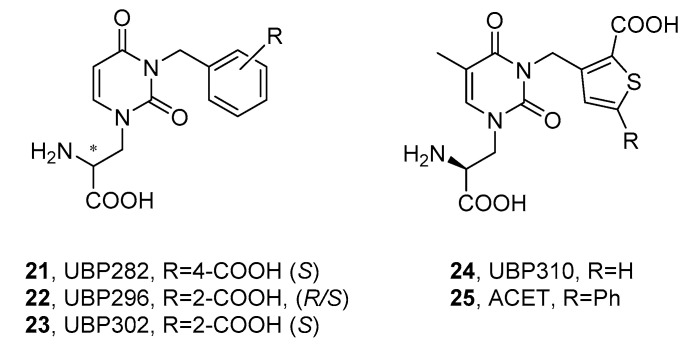
Structures of selected willardiine-based KAR antagonists. * the stereoisomeric center in the molecule.

A separate group of competitive AMPA/KAR antagonists includes compounds with α-amino acidic functionality, most often linked through a heterocyclic ring system with a distal acidic group; for example, a carboxylate or phosphonate, or their isostere, for example, tetrazolyl ring. Undoubtedly, the most important chemical class within this category is willardiine derivatives, developed based on the natural agonist of the AMPA/KA receptor, willardiine ((*S*)-1-(2-amino-2-carboxyethyl)pyrimidine-2,4-dione). One of the first attempts to achieve an antagonistic AMPAR/KAR profile among willardiine derivatives involved the introduction of a 4-carboxylbenzyl (UBP282, **21**) or 2-carboxybenzyl (UBP296, **22**) substituent at position *N*3 (Figure 4) [102,103]. Subsequent studies have shown that both UBP296 and its purified *S*-enantiomer UBP302 (**23**) proved to be potent and highly selective GluK1 antagonists (Table 3 and Table 4), with a negligible binding affinity at the homomeric rat GluK2, GluK5, or GluK2/GluK5 heteromers. UBP296 was further characterized on the native GluK1-containing receptors in the hippocampal mossy fibers and was found to play a role in controlling synaptic transmission in the CA1 and CA3 regions and reversibly block LTP induction in MF synapses [103].

Later findings showed that the benzene moiety could be successfully replaced with the tiophene ring (UBP310, **24**), resulting in a >500-fold selectivity for native KA receptors over AMPA and NMDA receptors expressed in neonatal rat motor neurons. Further studies on cloned homomeric KARs revealed a high selective affinity of **24** at the GluK1 and GluK3 receptors [104,105,106]. Interestingly, it was reported that, although glutamate-evoked currents mediated by recombinant GluK3 homomeric receptors were effectively blocked by UBP310 (with an IC_50_ of 4.0 μM), the compound did not block recombinant GluK2/3 heteromers and, unlike CNQX, it did not affect presynaptic kainate receptors in mouse hippocampal mossy fiber synapses, which are most likely composed of GluK2/3 heteromers. A similar behavior was observed for the compounds UBP302 (**23**) and ACET (UBP316, **25**) [23]. In addition, UBP310 demonstrated activity toward postsynaptic GluK2/5 receptors, inhibited slow excitatory postsynaptic currents (EPSC) mediated by KARs in epileptic mice, and was successfully tested in a mouse model of temporal lobe epilepsy [44].

The neuroprotective potential of UBP310 was studied in animal models of Parkinson’s disease (PD) [107]. Administration of this compound significantly improved the survival of the dopaminergic and total neuronal population in the substantia nigra pars compacta (SNpc) in the 1-methyl-4-phenyl-1,2,3,6-tetrahydropyridine (MPTP)-induced mouse model of PD. On the contrary, UBP310 lacked the ability to influence the MPTP-induced loss of dopamine levels or striatum dopamine transporter expression, and the deletion of GluK1, GluK2, or GluK3 appeared to have no effect on the MPTP- or UBP310-induced effects. Furthermore, **24** did not reduce the intracerebral cell loss induced by 6-OHDA intoxication. The results clearly suggested that the neuroprotective effects mediated by UBP310 in the midbrain were not related to its affinity for the specific kainate receptor subunits [107].

Recent studies have also revealed that UBP310, as an antagonist specific for the GluK1 subunit, was able to reduce the glutamate-induced desensitization of heteromeric GluK1/2 and GluK1/5 receptors. As the binding of an agonist by one subunit may be sufficient for receptor activation, but not sufficient for receptor desensitization, it is reasonable to believe that subunit-selective antagonists can be used as tools to efficiently reduce heteromeric receptor desensitization [108].

X-ray crystal structures solved for GluK1-LBD in complex with UBP302 and UBP310 have been used to design a close analogue of UBP310, ACET (UBP316, **25**), with a similar pharmacological profile. The ACET compound was applied to revisit the physiological role of kainate receptors in the NMDAR-independent mossy fiber LTP, as well as to establish the presynaptic regulation of Ca^2+^ facilitation in giant mossy fiber boutons (MFB). As a potent antagonist of GluK1-containing KARs (*K*_b_ approximately 1 nM), **25** was found to fully block LTP induction in the CA3-MF region of the hippocampus in a reversible manner, as well as to depress the presynaptic short-term facilitation of calcium transients in the MFB evoked by a train of action potentials [23,109].

So far, the crystal structures of the GluK1-LBD in complex with willardiine derivatives have been resolved for **23**, **24**, and **25** [104,109], all revealing an analogous binding mode of the ligand with residues of the LBD domains 1 and 2. In all cases, the carboxylic group of amino acid functionality was involved in the essential ion pair interaction with the side chain of Arg508 (the numbering of residues in the rat GluK1-LBD construct according to ref. [104]), as well as a hydrogen bond with the backbone amide group of Thr503. The α-amino group formed H-bond contacts with Thr503 and Pro501. Furthermore, for all three willardiine derivatives, a direct hydrogen bond interaction was observed between the distal carboxyl group and the amide group of Thr675.

**Table 3:** Activity of willardiine-derived antagonists at native AMPA and KA receptors in functional electrophysiological assays (all values in μM).

cmpd	Native iGluRs	
	AMPA IC_50_ *^a^*	GluK1-Containing KARs *K*_D_ *^b^*
UPB282 (**21**)	10 ± 2 *^c^*	9.3 ± 0.5 *^d^*
UBP296 (**22**)	98 ± 9 *^d^*	1.1 ± 0.1 *^d^*
UBP302 (**23**)	106 ± 13 *^d^*	0.40 ± 0.05 *^d^*
UBP310 (**24**)	16 ± 5 *^e^*	0.018 ± 0.004 *^e^*
ACET (UBP316, **25**)	17 ± 1 *^e^*	0.012 ± 0.001 *^e^*

*^a^* depression of the fast component of the dorsal root potential evoked ventral potential (fDR-VRP) in the spinal cord of a neonatal rat (a measure of antagonistic activity of AMPA receptors expressed on motoneurons); *^b^* native GluK1-containing receptors from the spinal cord of a neonatal rat; inhibition of kainate-induced depolarization in dorsal root fibers of a neonatal rat determined from the Gaddum–Schild equation; *^c^* ref. [110]; *^d^* ref. [103]; *^e^* ref. [105].

**Table 4:** Activity of willardiine-derived antagonists at recombinant homomeric AMPA and kainate receptors (all values in μM).

cmpd	Recombinant Homomeric iGluRs				
	GluK1	GluK2	GluK3	GluK5	GluA2
UPB282 (**21**) *^a^*	nd	>100 (IC_50_)	nd	>1000 (IC_50_)	nd
UBP296 (**22**)	0.60 ± 0.10 (*K*_b_) *^b^*	>1000 (IC_50_) *^a^*	374 ± 122 (*K*_i_) *^c^*	>100 (IC_50_) *^a^*	>300 (*K*_b_) *^b^*
UBP302 (**23**)	0.60 ± 0.10 (*K*_b_) *^d^*	>1000 (*K*_i_) *^c^* >300 (IC_50_) *^d^*	4.0 ± 0.2 (IC_50_) *^e^*	nd	>300 (IC_50_) *^d^*
UBP310 (**24**)	0.022 ± 0.005 (*K*_i_) *^f^* 0.010 ± 0.001 (*K*_b_) *^d^*	>100 (IC_50_) *^d^*	0.93 ± 0.12 (*K*_i_) *^f^* 0.023 ± 0.002 (IC_50_) *^e^*	>100 (*K*_i_) *^g^*	>100 (IC_50_) *^d^*
ACET (**25**)	0.0070 ± 0.0010 (*K*_b_) *^d^*	>100(IC_50_) *^d^*	0.092 ± 0.009 (IC_50_) *^e^*	nd	>100 (IC_50_) *^d^*

*^a^* ref. [103], radioligand binding assays to rat membranes using [^3^H]KA; *^b^* ref. [99], functional calcium fluorescence assays—blocking of 100 μM glutamate-induced calcium influx. The dissociation constant (*K*_b_) was determined from the IC_50_ value according to the Cheng–Prusoff equation; *^c^* ref. [99], radioligand binding assays to rat membranes using [^3^H]KA; *^d^* ref. [105], functional calcium fluorescence assays—blocking of 100 μM glutamate-induced calcium influx. The dissociation constant (*K*_b_) was determined from the IC_50_ value according to the Cheng–Prusoff equation; *^e^* ref. [23], functional electrophysiological assays—inhibition of glutamate-induced currents recorded in *Xenopus* oocytes expressing the receptors after injection of mRNA from rat cortex; *^f^* ref. [111], radioligand binding assays to rat membranes using [^3^H]AMPA, [^3^H]SYM2081; *^g^* ref. [13], radioligand binding assays to rat membranes using [^3^H]KA; nd—not determined.

#### 3.2.2. Decahydroisoquinolines

Decahydroisoquinoline derivatives, substituted in the direct vicinity of the nitrogen atom with a carboxylic acid group, can be treated as an expanded version of natural α-amino acid-based iGluR agonists such as glutamate or kainate, but with the α-amino group involved in the cyclic system. Compounds with this scaffold constitute a significant class of competitive AMPAR/KAR antagonists and usually possess a flexible linker with an attached distal acidic group, most often the tetrazolyl or carboxyl group.

**Figure 5:**
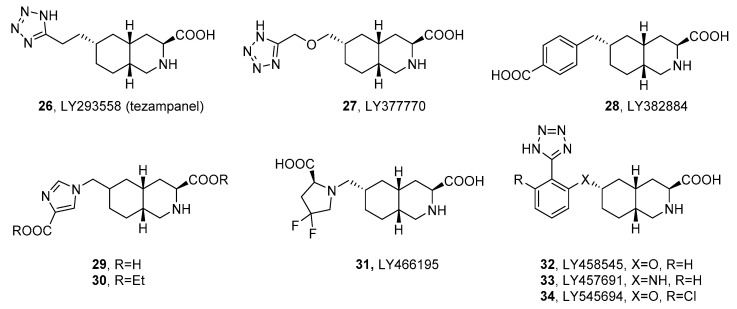
Structures of *cis*-decahydroisoquinoline-derived KAR antagonists.

One of the first compounds reported among this chemical class, acting as a competitive antagonist of AMPA/GluK1 receptors with micromolar affinity, was LY215490. Its stereoisomer LY293558 (**26**, tezampanel, Figure 5) was found to produce pain relief after oral surgery and in migraine patients and has undergone phase II clinical trials in patients with acute migraine [112,113,114]. Recent studies using rats exposed to soman also revealed its antiepileptic and neuroprotective efficacy after acute exposure to nerve agents and other organophosphate compounds both in monotherapy and in coadministration with other substances. In vivo studies with immature, young-adult, and aged rats exposed to soman demonstrated that **26** provided protection against brain impairment, prevented long-term behavioral deficits, and terminated status epilepticus, even with 1 h postexposure administration of tezampanel. In addition, full neuroprotection was achieved after simultaneous therapy with the NMDAR antagonist, caramiphen [115,116].

Competitive antagonists with improved selectivity for kainate receptors have been developed to enhance neuroprotective properties and efficacy in pain conditions with an improved side-effect profile [117]. The selective GluK1 antagonist LY377770 (**27**, Table 5) demonstrated neuroprotective effects in models of global and focal ischemia at doses of 80 mg/kg administered intraperitoneally (i.p.) followed by 40 mg/kg i.p. 3–6 h after the initial dose [118]. The compounds LY377770 and LY382884 (**28**) blocked epileptic activity in hippocampal slices and in vivo limbic seizures in conscious rats induced by pilocarpine or 6 Hz corneal stimulation [119]. The study of the effect on formalin-induced pain behavior demonstrated the antinociceptive efficacy of LY382884 without ataxia at doses of 5, 10, 30, and 100 mg/kg i.p. [120]. Meanwhile, in a primate model of peripheral neuropathy, **28** injected into the dorsal horn of the spinal cord via microdialysis (at a concentration of 100 μM–10 mM) reduced the nociceptive responses and in a concentration-dependent manner attenuated the response to mechanical stimuli [121].

The improvement in oral bioavailability was achieved by preparing a diethyl ester for the selective GluK1 receptor antagonist (**29**). The resulting prodrug (**30**) showed a high oral efficacy in two animal migraine models. In the neurogenic plasma protein extravasation (PPE) model, **30** administered orally (p.o.) 1 h before trigeminal stimulation exhibited efficacy with an estimated ID_50_ = 100 pg/kg, while, in the nucleus caudalis *c-fos* expression model, 1 h of pretreatment with a dose of 10 mg/kg resulted in an approximately 30% decrease in the central Fos expression. Furthermore, **29** in the PPE model administered intravenously (i.v.) 10 min before stimulation demonstrated efficacy with an estimated ID_50_ of 0.03 pg/kg [122].

A particularly interesting example of a potent and selective antagonist was LY466195 (**31**) [123,124], which has been found to have more than a 100-fold selectivity for GluK1 over other kainate and AMPA receptor subtypes. Both LY466195 and the diethyl ester prodrug showed significant inhibitory effects in two preclinical models of migraine (**31** administered i.v. and its ester administered p.o. in the PPE model exhibited an estimated ID_50_ of 100 μg/kg, while in the *c-fos* essay 31 demonstrated efficacy at doses of 1 to 100 μg/kg i.v. and its prodrug above a dose of 100 μg/kg). Compound **31** also demonstrated a lack of vasoconstrictive properties in the rabbit saphenous vein, suggesting that the vasoconstrictive properties of triptan molecules are not required for efficacy in PPE or *c-fos* assays [123].

Further improvement of the in vitro profile was achieved by the introduction of an oxygen linker and phenyl substituent in tetrazole derivatives. The most active compounds in the series, LY458545 (**32**) and LY457691 (**33**), showed a strong antagonism toward the AMPA and GluK1 receptors, but exhibited poor oral bioavailability. On the contrary, their prodrugs showed bioavailability levels of 24% and 41%, respectively, and both ester prodrugs showed oral efficacy in three animal models of pain. In the formalin test, prodrugs demonstrated efficacy at doses starting from 3 mg/kg and 5 mg/kg p.o., respectively. Furthermore, both compounds affected the dose-dependent reversal of carrageenan-induced thermal hyperalgesia with minimum effective doses (MED) of 1.0 mg/kg and 3.0 mg/kg p.o., and dose-dependent reverse capsaicin-induced mechanical hyperalgesia with MED of 1.0 and 10 mg/kg p.o., respectively [56].

Further enhancement of the selectivity and affinity for the GluK1 receptors was achieved by evaluating the structure of earlier tetrazole derivatives and introducing a chlorine substituent to obtain LY545694 (**34**). The 2-ethylbutyl ester of LY 545,694 showed a bioavailability of 42% in rats after oral administration and was shown to be effective in two animal models of persistent pain (dose-dependent reversal of carrageenan-induced thermal hyperalgesia with MED of 3 mg/kg p.o. and formalin model with MED of 1 mg/kg p.o.) [125]. The LY545694 tosylate has been studied in phase II of clinical trials for the treatment of pain caused by osteoarthritis of the knee (OA) and diabetic neuropathic pain (DPNP) [126].

The binding mode of the decahydroisoquinoline derivatives in the GluK1 receptor has been studied in the examples of the X-ray structures of the GluK1-LBD complexes with compounds **31** or **34** [124,125]. In both cases, the interactions of the ligand α-amino acid group involved in the isoquinoline system with the residues of the binding pocket resembled the canonical interactions previously observed for agonists and competitive antagonists with free α-amino acid functionality, e.g., willardiine derivatives.

**Table 5:** Receptor binding affinity of selected *cis*-decahydroisoquinoline derivatives at recombinant homomeric iGluRs, determined in radioligand binding assays (all values in μM).

cmpd	*K*_i_ [μM]			
	Recombinant Homomeric iGluRs			
	GluA2	GluK1	GluK3	GluK5
LY293558 (**26**) *^a^*	3.2 ± 0.3	4.2 ± 0.3	>100	>100
LY377770 (**27**) *^b^*	35 ± 6	3.1 ± 1.0	nd	nd
LY382884 (**28**) *^c^*	553	3.6	nd	nd
**29** *^d^*	117 ± 16	0.16 ± 0.08	49 ± 2	nd
LY466195 (**31**) *^e^*	269 ± 22	0.050 ± 0.020	8.9 ± 0.2	270 ± 31
LY458545 (**32**) *^f^*	8.3 ± 2.0	1.7 ± 0.4	nd	nd
LY457694 (**33**) *^f^*	5.5 ± 1.8	1.6 ± 0.6	nd	nd
LY545694 (**34**) *^g^*	30	0.2	nd	nd

*^a^* ref. [100], radioligands: GluA2, [^3^H]AMPA; GluK1,3,5, [^3^H]KA; *^b^* ref. [127], radioligands: GluA2, [^3^H]AMPA; GluK1,3, [^3^H]KA *^c^* ref. [117], radioligands: GluA2, [^3^H]AMPA, GluK1, [^3^H]ATPA; *^d^* ref. [122], radioligands: GluA2, [^3^H]AMPA; GluK1,3, [^3^H]KA; *^e^* ref. [123], radioligands: GluA2, [^3^H]AMPA; GluK1,3,5, [^3^H]KA; *^f^* ref. [56], radioligands: GluA2, [^3^H]AMPA; GluK1, [^3^H]KA; *^g^* ref. [125], radioligands: GluA2, [^3^H]AMPA, GluK1, [^3^H]ATPA; nd—not determined.

#### 3.2.3. Other α-Amino Acid Antagonists

**Figure 6:**
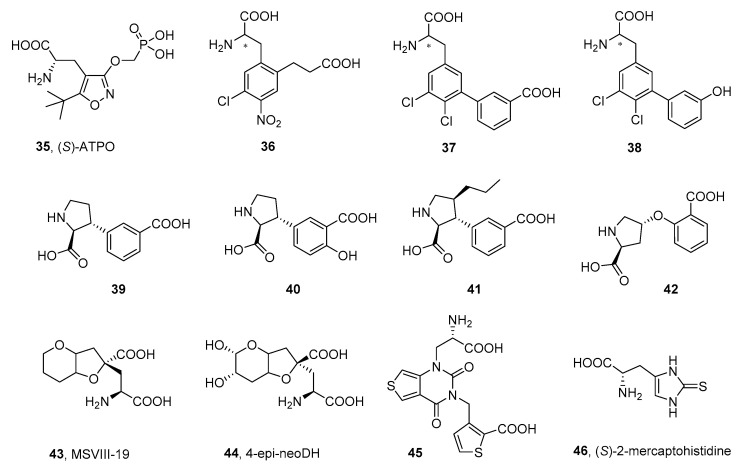
Structures of selected α-amino acid antagonists of kainate receptors. * the stereoisomeric center in the molecule.

The good example of this group are α-amino acids designed as expended versions of the natural iGluR agonist AMPA, with a structure based on the isoxazole core. Compounds in this series were designed primarily as competitive antagonists of the AMPA receptor (see the reviews of Nikam [83] and Catarzi [82]), and studies often lack data on the affinity at the kainate receptors. In this regard, one of the most developed compounds in this group is (*S*)-ATPO (**35**), the selective AMPAR/GluK1 antagonist that shows an affinity in the low micromolar range for the homomeric GluK1 receptors (Figure 6, Table 6) [128,129]. Analysis of the X-ray structure of ATPO in complex with GluK1 revealed that, in addition to the typical binding mode of the α-amino acid group, seen for other α-amino acid agonists and antagonists in GluK1- or GluA2-LBD, (*S*)-ATPO also interacts with its phosphonate tail, forming hydrogen bonds with the non-conserved serine of GluK1, which probably accounts for the observed selectivity profile of the compound [130].

Furthermore, the isoxazole ring of (*S*)-ATPO does not form any specific interactions with the receptor, acting mainly as a spacer between the α-amino acid group and the phosphonate functionality. Therefore, the structure of (*S*)-ATPO became an inspiration for a series of phenylalanine-based AMPAR/KAR antagonists, in which the isoxazole spacer has been replaced by the benzene ring, substituted with the 2-carboxyethyl chain (**36**) [131,132]. The antagonist **36** showed an equimolar affinity at the native AMPA receptors and homomeric GluK1 receptors in the range of 3 μM and was cocrystallized with the AMPA subunit GluA2-LBD, as well as with the kainate subunit GluK1-LBD, inducing in both X-ray complexes a domain closure similar to that observed in the iGluR structures with partial agonists. The binding mode of **36** for both receptors resembled the binding modes of the (*S*)-ATPO and willardiine-based antagonists, with the canonical interaction pattern between the α-amino acid group and the arginine, proline, and threonine residues of the domain 1, conserved among all the non-NMDA subunits. Furthermore, an additional interaction between the nitro group and the Ser721 side chain (which, together with Glu441, forms the so-called ‘interdomain lock’ stabilizing the active state of the receptor), was suggested as an important determinant of ligand affinity.

Another series of phenylalanines reported by the same group was based on the biphenylalanine core substituted with the distal acidic group, either carboxy or phenolic hydroxy substituent (**37**, **38**) [133,134,135]. Interestingly, it appeared that this distal polar group had a strong influence on the iGluR affinity profile: compounds with a 3-carboxy group attached to the distal phenyl ring (**37**) preferentially bound to GluK1 receptors (with a 35-fold selectivity over AMPAR in the case of **37**), while the 3-hydroxy group at this position was optimal for binding to native AMPA receptors (**38**) (with a selectivity of 21 times over GluK1 receptors in the case of **38**).

The structure of kainic acid became an inspiration for the design of a series of proline derivatives that contained an additional carboxyl group in their structure. The first structure described in this series, **39**, demonstrated a micromolar affinity at the homomeric GluK1 receptor with a 20-fold selectivity over the homomeric GluK2 and GluK3 receptors (Table 4) [136]. Further modifications of the structure resulted in the improved selectivity and affinity at the homomeric GluK1 receptors (**41**, **42**) [111,137], while, in the case of **40**, the resulting compound exhibited a 5-fold increased affinity at the GluK3 subunit compared to the GluK1 subunit [137,138,139]. The X-ray structures of the GluK1-LBD cocrystallized with **41** or **42** were successfully resolved, which allowed for determining the binding mode of the proline derivatives in the binding pocket [111,137]. The α-amino acid moiety of the ligands interacted with the LBD residues in a manner similar to that described above.

Dysiherbaine derivatives belong to a group of active KAR ligands originally isolated from the marine sponge *Lendenfeldia chodrodes*. The synthetic derivative MSVIII-19 (**43**) was the first to demonstrate strong and selective antagonistic properties toward GluK1 homomeric receptors, with no affinity for GluA2 receptors [140,141,142]. Compound **43** showed analgesic activity in animal model studies, supporting the thesis of the potential of GluK1 antagonists in the treatment of pain [143]. The structural variants of neodysiherbaine (**44**) [144] did not show an improved affinity and selectivity for kainate receptors, compared to **43**, but led to useful tools for studying the structure of KAR subunits [145].

In the group of bicyclic pyrimidine-2,4-dione analogs, on the other hand, subsequent structural adjustments have resulted in compound **45**, which exhibited a remarkable selectivity for kainate receptors, especially the GluK1 subunit (over a 400-fold higher affinity for GluK1 compared to GluA2 and 53 times versus GluK3). Functional studies confirmed the antagonist profile of **45**, while the in vivo mouse formalin test of prolonged acute pain stimulation proved its analgesic efficacy at a dose of 2 mg/kg [146]. The molecular interactions observed in the crystal structure resolved for **45** in complex with the GluK1-LBD correspond to those previously observed for other willardiine derivatives [147]. In particular, the carboxyl group of the amino acid moiety forms an ion pair interaction with the side chain of Arg523 (Arg508 according to residue numbering in ref. [104]), and a hydrogen bond with the backbone of Thr518 (Thr503), while the α-amino group is involved in multiple H-bond contacts with the carbonyl group of Pro516 (Pro501), the side chain of Thr518, and, additionally, with the Glu738 of domain 2. On the other hand, the flexible carboxythienyl part of molecule **45** interacts with the residues of domain 2 of the GluK1-LBD, thus stabilizing an open conformation of the binding pocket [147].

(*S*)-mercaptohistidine (**46**) represents one of the first selective GluK3 receptor antagonists described, with a 30-fold affinity preference over GluK1, GluA3, and GluA4 and a selectivity of more than 100-fold over the recombinant receptors GluK2, GluA1, and GluA2, determined in radioligand binding assays (Table 6). Surprisingly, the functional evaluation revealed antagonistic activity for the GluK3 receptors, but also weak agonist activity for the GluA2 receptors, making **46** the first orthosteric iGluRs ligand with a mixed agonist–antagonist profile for KARs/AMPARs [148].

**Table 6:** Receptor binding affinity of selected α-amino acids antagonists at native and recombinant homomeric iGluRs, determined in radioligand binding assays (all values in μM).

cmpd	IC_50_/*K*_i_ [μM]		*K*_i_ [μM]			
	Native iGluRs		Recombinant Homomeric iGluRs			
	AMPA [^3^H]AMPA	KA [^3^H]KA	GluK1	GluK2	GluK3	GluA2
**35**	16 ± 1 (IC_50_) *^a^*	>100 (IC_50_) *^a^*	2.2 ± 0.5 *^b^* 2.9 ± 0.3 *^c^* 2.6 ± 0.4 *^d^*	>100 *^c,d^*	>100 *^c^* >1000 *^d^*	60 ± 5 *^d^*
**36** *^e^*	3.4 ± 0.3 (IC_50_)	nd	3.0 ± 0.8	nd	14 ± 3	nd
(*S*)-**36** *^e^*	nd	nd	1.5 ± 0.2	nd	8.0 ± 1.1	nd
**37** *^c^*	>100 (IC_50_)	>100 (IC_50_)	2.8 ± 0.8	>100	>100	nd
**38**	4.6 ± 0.2 (IC_50_) *^c^*	>100 (IC_50_) *^c^*	92 ± 2 *^f^*	78 ± 10 *^f^*	52 ± 3 *^f^*	5.0 ± 0.4 *^f^*
**39** *^g^*	51 ± 10 (IC_50_)	22 ± 2 (IC_50_)	4.3 ± 0.4	>100	8.1 ± 0.6	67 ± 16
**40** *^d^*	2.0 (IC_50_)	1.4 (IC_50_)	4.8 ± 1.5	10–100	0.87 ± 0.09	nd
**41** *^d^*	48 (IC_50_)	22 (IC_50_)	0.62 ± 0.10	81 ± 8	2.2 ± 0.4	nd
**42** *^h^*	>100 (*K*_i_)	>100 (*K*_i_)	4.0	>100	>100	nd
**43**	nd	nd	0.13 ± 0.02 *^i^*	>100 *^i^*	>100 *^i^*	>100 *^j^*
**44**	nd	nd	2.4 *^j^* 0.56 *^k^*	7.7 *^j^* 6.7 *^k^*	>100 *^k^*	>100 *^j^* >30 *^k^*
**45** *^l^*	nd	nd	0.16 ± 0.02	99 ± 22	7.5 ± 0.3	73 ± 15
**46** *^m^*	>100 (*K*_i_)	>100 (*K*_i_)	177 ± 4	>5000	6.4 ± 0.7	>1000

*^a^* ref. [129]; radioligands: [^3^H]AMPA, [^3^H]KA; *^b^* ref. [130], affinity given for (*R*,*S*)-ATPO, radioligand: [^3^H]SYM2081; *^c^* ref. [133], radioligand: GluK1-3, [^3^H]SYM2081; *^d^* ref. [111], radioligands: GluA2, [^3^H]AMPA, GluK1-3, [^3^H]SYM2081; *^e^* ref. [132], radioligand: GluK1,3, [^3^H]SYM2081; *^f^* ref. [134], radioligands: GluA2, [^3^H]AMPA, GluK1-3, [^3^H]SYM2081; *^g^* ref. [136], radioligands: GluA2, [^3^H]AMPA, GluK1-3, [^3^H]SYM2081; *^h^* ref. [137], radioligands: GluK1, [^3^H]-(*S*)-NF608, GluK2-3, [^3^H]KA; *^i^* ref. [140], radioligand: GluK1-3, [^3^H]KA; *^j^* ref. [145], radioligands: GluA2, [^3^H]AMPA, GluK1-2, [^3^H]KA; *^k^* ref. [144], radioligands: GluA2, [^3^H]AMPA, GluK1-2, [^3^H]KA; *^l^* ref. [147], radioligands: GluA2, [^3^H]AMPA, GluK1-3, [^3^H]SYM2081; *^m^* ref. [148], radioligands: GluA2, [^3^H]AMPA, GluK1, [^3^H]KA or [^3^H]-(*S*)-NF608, GluK2-3, [^3^H]KA; nd—not determined.

### 3.3. Structurally Dissimilar AMPAR/KAR Antagonists

**Figure 7:**
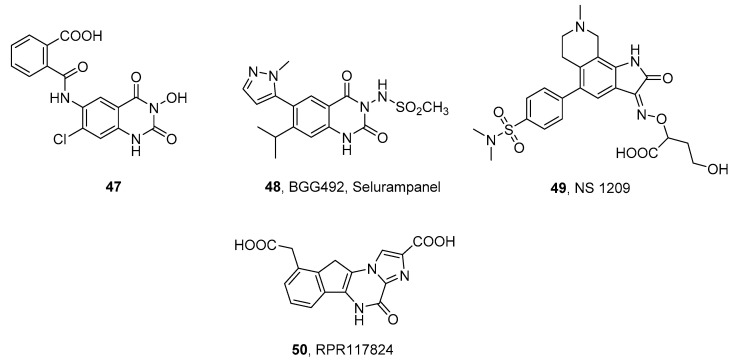
Structurally dissimilar AMPAR/KAR antagonists.

In addition to the chemical classes described above, some structurally dissimilar competitive antagonists of AMPA/KA receptors with in vivo anticonvulsant activity have been identified [149], including quinazoline-2,4-diones (**47** and BGG492, selurampanel, **48**) [150,151], isatin oximes (NS 1209, **49**) [152,153], and derivatives of pyrazine (RPR117824, **50**, Figure 7, Table 7) [154,155]. NS 1209 showed potent and dose-dependent anticonvulsant activity in various preclinical models and alleviated refractory status epilepticus and neuropathic pain in phase II of clinical trials, but further research on the molecule has been suspended [153,156]. RPR117824 in in vivo models blocked convulsions induced in mice or rats by supramaximal electroshock or chemoconvulsive agents such as pentylenetetrazol and showed significant neuroprotective activity in global and focal cerebral ischemia and brain and spinal cord trauma [154,155]. In the group of quinazoline-2,4-dione derivatives, compound **47** showed a high affinity for native KA receptors and demonstrated neuroprotective effects in an in vitro model of cerebral ischemia [157,158]. BGG 492 (selurampanel), an orally active AMPAR/KAR antagonist (the functional efficacy on the rat cortical wedge for the AMPAR and KAR was 0.46 and 0.42 μM, respectively), has shown anticonvulsant activity in animal models of epilepsy, such as electroshock or chemically induced seizures in rodents. Currently, **48** has been subjected to clinical trials aimed at patients with epilepsy, tinnitus, and migraine [150].

## 4. Non-Competitive Antagonists and Channel Blockers of Kainate Receptors

Despite the fact that the pharmacology of non-competitive inhibitors of AMPA and kainate receptors remains very similar, AMPAR antagonists have attracted more interest in recent years, especially as antiepileptic agents. Among them, a certain number of compounds likely act as non-selective AMPAR/KAR inhibitors, and only a few examples of selective kainate receptor non-competitive antagonists have been described.

**Figure 8:**
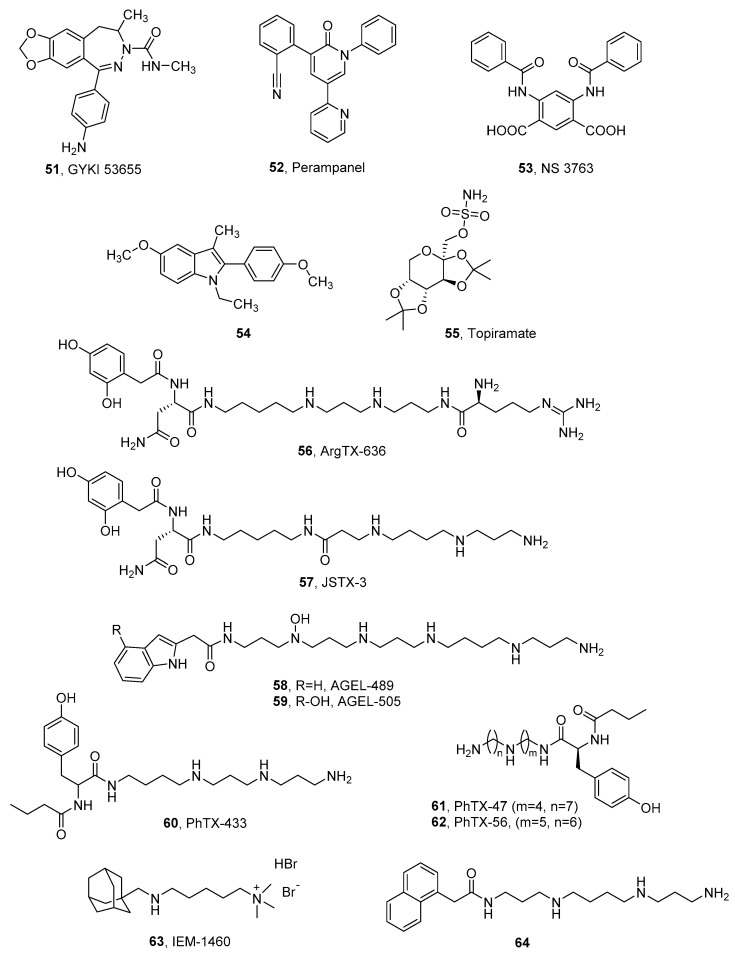
Non-competitive antagonists and channel blockers of kainate receptors.

The early described group of non-competitive antagonists of non-NMDA receptors, 2,3-benzodiazepine derivatives, are widely recognized as selective AMPAR antagonists with very low potency and efficacy for kainate receptors. Considering that members of this chemical class selectively block AMPAR-conducted responses, some of them are used as pharmacological tools to isolate the corresponding neuronal response from kainate receptors. The compound GYKI 53,655 (**51**, Figure 8, Table 8) was shown not to affect GluK1 or GluK2-containing kainate receptors or native somatodendritic kainate receptors (IC_50_ > 200 mM) [16,23,160]. However, in the case of GluK3-containing receptors expressed in HEK 293 cells, GYKI 53,655 was able to block currents from activated GluK3 homomeric receptors with an IC_50_ = 63 ± 10 µM, and from GluK2/3 heteromeric receptors with an even better result, IC_50_ = 32 ± 5 µM. Furthermore, at high concentrations, the compound was found to block presynaptic kainate receptors in mossy fiber synapses (likely composed of GluK2/3 heteromers) and decrease short-term plasticity [23].

A real breakthrough in iGluR research was the development of perampanel (**52**, Fycompa, Figure 8), approved as an oral drug for partial-onset seizures and generalized primary tonic–clonic seizures [164]. Initial studies showed that **52** inhibits native AMPA receptors and also individual AMPAR subunits with no affinity for NMDARs and KARs [165]. However, recent structural studies of AMPARs in complex with perampanel have shown that its binding site consists of amino acids that are also highly conserved for the GluK4 and GluK5 receptor subunits. Consequently, contrary to previous assumptions, **52** demonstrated the effective inhibition of the heteromeric GluK1/5 and GluK2/5 receptors at levels comparable to the native AMPA receptors (Table 9). Furthermore, the presence of NETO1 and NETO2 was shown to significantly affect KAR affinity, particularly heteromers containing the GluK2 subunit (Table 9) [166]. Perampanel has been included in a wide range of clinical trials for epilepsy among children and adults [164,167,168,169,170,171,172,173,174,175], as well as in studies for Parkinson’s disease [176,177] or amyotrophic lateral sclerosis [178,179] (Table 10).

The non-selective inhibitory effect of kainate receptors was also observed in the case of *cis*-unsaturated fatty acids. In contrast to fully saturated fatty acids, docosahexaenoic acid (DHA), arachidonic acid (AA), linolenic acid, and linoleic acid demonstrated a significant reduction in kainate-induced whole cell currents and reversibly inhibited kainate receptor signaling by acting within the TMD [180,181,182].

A group that has contributed to a better understanding of the physiology and pathophysiology of kainate receptors [183,184] is arylureidobenzoic acids, in particular 4,6-bis (benzylamine)-1,3- benzenedicarboxylic acid (NS 3763, **53**), which is a selective antagonist of the homomeric GluK1 receptor, lacking an affinity for the native AMPAR and GluK1/2 and GluK1/5 heteromers [162,183,185,186]. Proceeding with research on selective kainate receptor inhibitors, the group of Kaczor et al. reported 1,2,3,5-tetrasubstituted indole derivatives, including the first non-competitive GluK2 receptor ligand known so far (**54**) [163]. Studies on these derivatives have contributed to the development of computational research on the structure of kainate receptor subunits and ligand binding sites [163,187,188].

A drug whose exact mode of action has not yet been clarified is topiramate (**55**). Its potent neuroprotective and anticonvulsant effect has been confirmed by several studies using animal models, and at least three mechanisms appear to contribute to this action profile: (1) enhancement of the GABA response; (2) blocking voltage-dependent ion channels, and (3) inhibition of AMPA/KA receptors [189]. Meanwhile, topiramate has been shown to be ineffective against AMPA- or NMDA-induced clonic seizures, but it is effective in blocking kainate-induced seizures [190,191]. Subsequent in vivo studies investigating the blocking of the agonist-induced seizure activity of iGluRs confirmed that topiramate selectively inhibits the GluK1 subunit response [190,192]. For its unique properties, topiramate is used as a treatment for epilepsy with partial and generalized tonic–clonic seizures, as well as for migraine prevention and treatment. In addition to this, several clinical trials have been conducted for the treatment of eating disorders, obesity, alcohol dependence, neuropathy, post-traumatic stress disorder, and Tourette syndrome [38].

Inhibition of iGluR activity could also occur as a result of the binding of ion channel blockers. Among this group of derivatives, many naturally occurring compounds, as well as their synthetic analogs, have been described. All shared structural similarity (that is, polyamine moiety) and, when applied, blocked the AMPAR/KAR in a voltage-independent manner [193]. Within the KAR family, the GluK3 subunit demonstrated significant sensitivity to polyamines, especially compared to the GluK1 and GluK2 subunits. Meanwhile, auxiliary proteins of the kainate receptors, NETO1 and NETO2, attenuated the blocking of ion channels [194]. As mentioned above, permeability to Ca^2+^ as well as an affinity of polyamines is related to the editing of the Q/R site, where the kainate receptors of the “R” type are not permeable to Ca^2+^ and show no sensitivity to polyamines [16,17,195]. This effect was considered to derive from the electrostatic repulsive effect of positively charged arginine in the narrowest region of the pore. GluK4- and GluK5-containing heteromers have also been found to weaken polyamine blocking due to the presence of a proline residue that is not represented in the GluK1-3 subunits and changes the dynamics of the α-helical region of the selectivity filter [2,194,196,197].

Studies using the toxin of the spider venom *Argiope lobata* (ArgTX-636, **56**) [195], the *Joro spider* toxin (JSTX-3, **57**) [196], the agatotoxin of the North American funnel web spider *Ageleonopsis aperta* (AGEL-489, **58** and AGEL-505, **59**) [198], or the active venom fraction of *Philanthus triangulum* (PhTX-433, **60**) [199] demonstrated that polyamines are not only potent antagonists of ionotropic glutamate receptors, but can also exhibit other biological activities [200,201,202,203,204,205]. Synthetic derivatives of philanthotoxins allowed for determining structure–activity relationships, principally concerning AMPA receptors. The modification that resulted in an active blocking of the GluK1 subunit (PhTX-47, **61**, *K*_i_ = 0.060 ± 0.014 μM) involved altering the length of the polyamine chain with respect to the number of amine groups and the methylene bridges located between them. In contrast, even a slight modification of the chain length shifted activity at the GluA1 receptors (PhTX-56, **62**) with affinity at the GluA1 subunits of *K*_i_ = 3.3 ± 0.78 nM [203].

Other synthetic polyamine derivatives, IEM-1460 (**63**) [206] and *N*^1^-naphthylacetylspermine (**64**) [207], predominantly affect the GluA2-deficient Ca^2+^ receptors and further studies have shown that modifications of both the polyamine chain and the head group can contribute to changes in activity and selective affinity at the AMPA, KA, and NMDA receptors [208,209,210,211].

## 5. RNA Aptamers

**Figure 9:**
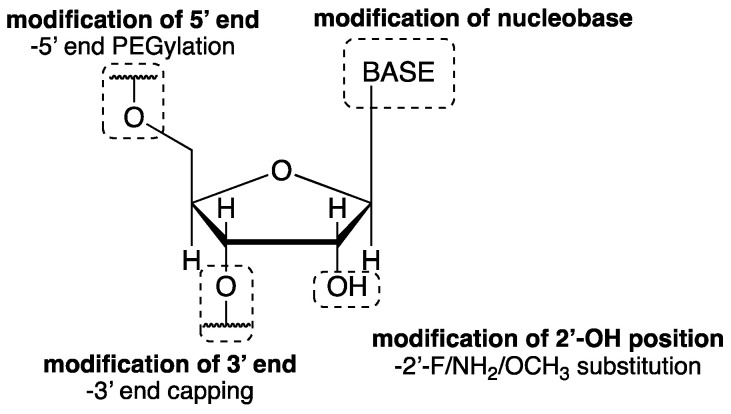
Common strategies in chemical modifications of RNA aptamers (adapted from ref. [217]).

A new approach to the development of non-competitive AMPA/KA receptor antagonists involves research on RNA aptamers, RNA molecules that affect the function of a biological target by binding to it similarly to how an antibody binds to an antigen with high affinity and specificity. Aptamers are identified using an RNA library by the systematic evolution of ligands through exponential enrichment (SELEX). Currently, aptamers are being used successfully as diagnostic and therapeutic tools in the treatment of cancers or virus infections. As potential drug candidates, they possess unique properties such as good water solubility, high activity, and selectivity. The limitations associated with the application of RNA aptamers are mainly related to their limited stability in vivo, due to the fact that they undergo rapid ribonuclease-catalyzed degradation and excretion by renal filtration [212]. Natural RNA undergoes degradation at the 2′-OH position of the nucleoside, with the half-life (t_1/2_) of unmodified aptamers typically lasting a few minutes. Chemical modifications aimed at improving RNA stability and that can extend t_1/2_ up to several days have included the replacement of the 2′-OH group with 2′-fluoro (2′-F), 2′-OCH_3_ or 2′-NH_2_ group, capping of the 3′-end, or conjugation with polyethylene glycol (PEG) (Figure 9) [212,213,214,215,216].

Among the first representatives of RNA aptamers that demonstrated activity toward non-NMDA receptors were FN1040 (**65**) and FN1040s (**66**) described by Huang et al. [215,218]. To identify the dominant sequence (FN1040), a mixture of competitive and non-competitive AMPA receptor antagonists with known mechanisms of action was used during the SELEX experiment, and the structural modification involved replacing 2′-OH with 2′-F. The FN1040s (69-nucleotide, nt) aptamer was a trimmed derivative of FN1040 (101-nt) designed to further improve stability and simplify production and was created by removing all non-essential nucleotides. Both aptamers exhibited activity at the ionotropic receptors and showed a high resistance to ribonuclease in the cerebrospinal fluid environment. The full-length FN1040 was active toward the GluA1, GluA2, GluK1, and GluK2 subunits, as well as GluN1a/2A and GluN1a/2B. Its shorter version, FN1040s, selectively inhibited the GluA1 and GluA2 subunits [215].

Jaremko et al. applied a similar strategy in the search for selective aptamers [216,219]. The full-length leading structure-AB9 (**67**, 101-nt) had the ability to selectively inhibit the GluA1 and GluA2 subunits. On the other hand, its truncated version, AB9s (**68**, 55-nt), inhibited both the AMPA and kainate receptors (GluK1 and GluK2 subunits) [219]. Based on 68, two mutants were created, named AB9s-r (**69**) and AB9s-b (**70**), respectively. As expected, both mutants showed no activity against NMDARs. AB9s-r favored the AMPA receptor subunits, while AB9s-b inhibited the homomeric GluK1-3 receptors, as well as the heteromers containing the GluK5 subunit. The next step, chemical modification involving the introduction of 2′-F groups, led to the corresponding derivatives: FBs-r (**71**) and FBs-b (**72**), and both exhibited much higher stability than their precursors, but maintained the same desired selectivity [216].

The exact binding site of RNA aptamers is still not resolved, but studies of homologous binding and registration throughout the cell suggest that the reported RNA aptamers most likely bind to the regulatory site of non-NMDA receptors and inhibit them in a non-competitive manner [219].

## 6. The Therapeutic Potential of Non-NMDA Receptors Antagonists

There is no doubt that glutamate ionotropic receptors have received great interest from researchers. In recent years, a variety of potent non-NMDAR antagonists have been synthesized and described by both academic researchers and pharmaceutical companies. Numerous of the described antagonists were found to be particularly useful in the context of their potential use in neurological diseases and have been studied both preclinically and clinically (Table 10), targeting conditions such as epilepsy, neuropathic pain, Parkinson’s disease, and migraine. Unquestionably groundbreaking was the launch of perampanel, a non-competitive AMPAR/KAR antagonist, as a treatment for partial-onset seizures with/without secondary generalized seizures and primary generalized tonic–clonic seizures.

**Table 10:** Competitive and non-competitive AMPAR/KAR antagonists that have entered clinical trials in the last 15 years [38].

Disease	Target/Mechanism	Drug	Stage of Development
Neuropathic Pain	Competitive GluK1 antagonist	LY 5,454,694 tosylate	Phase II (compl. 2010)
	Competitive AMPAR/GluK1 antagonist	NGX426 (tezampanel prodrug)	Phase I (compl. 2008)
	Competitive AMPAR/KA antagonist	NS 1209	Phase II (compl. 2007)
	Competitive AMPAR/GluK1 antagonist	Tezampanel (NGX424 LY 293558)	Phase I
Migraine	Competitive AMPAR/GluK1 antagonist	Tezampanel	Phase II (compl. 2009)
	Competitive AMPAR antagonist	Selurampanel	Phase II (compl. 2012)
	Non-competitive KAR antagonist	Topiramate	Phase IV (compl. 2015)
Epilepsy	Competitive AMPAR antagonist	Becampanel	Phase II (compl. 2011)
	Competitive AMPAR antagonist	Selurampanel	Phase II (compl. 2012)
	Competitive AMPAR antagonist	NS 1209	Phase II
	Non-competitive KAR antagonist	Topiramate	Phase IV (compl. 2010)
	Non-competitive AMPAR/KAR antagonist	Perampanel	Marketed 2012
	Non-competitive AMPAR antagonist	Talampanel	Phase II (compl. 2006)
Stroke and Head Trauma	Competitive AMPAR antagonist	ZK200775	Phase II (dis)
Amyotrophic Lateral Sclerosis	Non-competitive AMPAR antagonist	Talampanel	Phase II (compl. 2010)
	Non-competitive AMPAR/KAR antagonist	Perampanel	Phase II (terminated 2021)
Parkinson’s Disease	Non-competitive AMPAR antagonist	Talampanel	Phase II (compl. 2006)
	Non- competitive AMPAR/KAR antagonist	Perampanel	Phase III (compl. 2012)
Eating Disorder	Non-competitive KAR antagonist	Topiramate	Phase I (compl. 2018)
Obesity	Non-competitive KAR antagonist	Topiramate	Phase II (compl. 2017)
Chronic Subjective Tinnitus	Competitive AMPAR antagonist	Selurampanel	Phase II (compl. 2012)
Tourette Syndrome	Non-competitive KAR antagonist	Topiramate	Phase III (compl. 2008)
Glial Brain Tumors	Non-competitive AMPAR/KAR antagonist	Perampanel	Phase IV (compl. 2017)
	Non-competitive AMPAR antagonist	Talampanel	Phase II (compl. 2011)
Alcohol Dependence	Non-competitive KAR antagonist	Topiramate	Phase III (compl. 2011)

The patent review suggests a range of new potential uses for the described kainate and AMPA receptor antagonists. The application of compounds with this profile in combination with active NMDAR antagonists could be used to treat epilepsy, post-traumatic stress disorder, depression, brain injury, or anxiety [220,221]. On the other hand, the invention that involved the combined or sequential administration of NBQX and Gly-Pro-Glu tripeptide (GPE) as a modulatory agent suggested its potential in the prevention and treatment of CNS damage in mammals caused by demyelinating diseases, including multiple sclerosis [222].

Other possible applications of the simultaneous delivery of AMPAR/KAR and NMDAR antagonists could also be aimed at reducing the adverse side effects and limiting the harmful effects on the central nervous system [223]. Further implementations include targeted therapy for conditions such as cancer [224], obesity [225], mental illness [226], or withdrawal symptoms [227].

## 7. Conclusions

The studies presented in the above work strongly suggest that kainate receptors have an integral role in signaling at multiple levels of the CNS. However, despite the recent growth in knowledge in this field, many important issues remain unsolved, also due to the lack of appropriate selective pharmacological tools. The number of kainate receptor inhibitors with high activity and good results in preclinical studies has been reported; nevertheless, there is still room to improve the search for suitable drug candidates. Examples include numerous clinical trials that were terminated prematurely or discontinued, such as for the compounds NBQX or LY293558. New discoveries in the field of selective KAR antagonists or the launch of perampanel give reason for optimism and hope that, in the near future, we will better understand the role that KARs play in CNS development and diseases.

## Figures and Tables

**Table 7:** Receptor binding affinity of selected structurally dissimilar antagonists at native and recombinant homomeric iGluRs, determined in radioligand binding assays (all values in μM).

cmpd	Native iGluRs		Recombinant Homomeric iGluRs		
	AMPA	KA	GluA2	GluK1	GluK3
**47** *^a^*	16 (*K*_i_)	0.62 (IC_50_)	nd	nd	nd
**48**, selurampanel *^b^*	0.19 (IC_50_)	>100 (IC_50_)	nd	nd	nd
**49**, NS 1209 *^c^*	0.043 (IC_50_) *^c^*	81 (IC_50_) *^c^*	0.030 (*K*_i_) *^d^*	0.74 (*K*_i_) *^d^*	nd
**50**, RPR117824 *^e^*	0.018 (IC_50_)	nd	nd	nd	nd

*^a^* ref. [157], radioligands: [^3^H]AMPA, [^3^H]KA; *^b^* ref. [151], radioligands: [^3^H]CNQX, [^3^H]KA; *^c^* ref. [152,159] [^3^H]AMPA, [^3^H]KA; *^d^* ref. [152,159], radioligands: [^3^H]AMPA, [^3^H]SYM2081; *^e^* ref. [154], radioligand: [^3^H]AMPA; nd—not determined.

**Table 8:** Functional data for non-competitive antagonists of kainate receptors (all values in µM).

cmpd	IC_50_ [μM]			
	Recombinant Homomeric iGluRs			
	GluA1	GluA2	GluK1	GluK2
**51**, GYKI 53655	5.9 ± 0.1 *^a^*	1.9 ± 0.8 *^b^*	>100 *^b^*	198 ± 53 *^b^*
**53**, NS 3763 *^c^*	nd	nd	1.6 ± 0.2	>30
**54** *^d^*	nd	nd	4.0	0.7

*^a^* ref. [161], functional electrophysiological patch-clamp assay—inhibition of AMPA-induced currents recorded in human HEK293 cells transfected with GluA1 receptors; *^b^* ref. [23], functional electrophysiological assays—inhibition of glutamate-induced currents recorded in *Xenopus* oocytes expressing the receptors after injection of mRNA from rat cortex; *^c^* ref. [162], functional electrophysiological assays—inhibition of domoate-induced increase in intracellular calcium concentration in HEK293 cells transfected with homomeric GluK1 and GluK2 receptors; *^d^* ref. [163], functional calcium fluorescence assays—blocking of glutamate-induced calcium influx; nd—not determined.

**Table 9:** IC_50_ values for perampanel inhibition in voltage-clamp recordings from recombinant homomeric and heteromeric KARs expressed in HEK293-T/17 cells (adapted from ref. [166]).

Receptor	IC_50_ [μM]
GluA4	0.56 ± 0.12
GluK1	19 ± 5
GluK2	26 ± 10
GluK3	41 ± 6
GluK1/5	2.8 ± 0.3
GluK2/5	0.85 ± 0.09
GluK3/5	14 ± 8
GluK1/NETO1	11 ± 4
GluK1/NETO2	19 ± 4
GluK2/NETO1	5.4 ± 0.9
GluK2/NETO2	5.8 ± 1.0
GluK1/5/NETO2	0.99 ± 0.15
GluK2/5/NETO1	0.69 ± 0.16

## Data Availability

Not applicable.
